# High rate of virological failure and low rate of switching to second-line treatment among adolescents and adults living with HIV on first-line ART in Myanmar, 2005-2015

**DOI:** 10.1371/journal.pone.0171780

**Published:** 2017-02-09

**Authors:** Nang Thu Thu Kyaw, Anthony D. Harries, Ajay M. V. Kumar, Myo Minn Oo, Khine Wut Yee Kyaw, Than Win, Thet Ko Aung, Aung Chan Min, Htun Nyunt Oo

**Affiliations:** 1 International Union Against Tuberculosis and Lung disease, Mandalay, Myanmar; 2 International Union against Tuberculosis and Lung Disease, Paris, France; 3 London School of Hygiene and Tropical Medicine, London, United Kingdom; 4 International Union Against Tuberculosis and Lung disease, The Union South-East Asia Regional Office, New Delhi, India; 5 National HIV/AIDS program, Mandalay, Myanmar; 6 National HIV/AIDS program, Nay Pyi Taw, Myanmar; Azienda Ospedaliera Universitaria di Perugia, ITALY

## Abstract

**Background:**

The number of people living with HIV on antiretroviral treatment (ART) in Myanmar has been increasing rapidly in recent years. This study aimed to estimate rates of virological failure on first-line ART and switching to second-line ART due to treatment failure at the Integrated HIV Care program (IHC).

**Methods:**

Routinely collected data of all adolescent and adult patients living with HIV who were initiated on first-line ART at IHC between 2005 and 2015 were retrospectively analyzed. The cumulative hazard of virological failure on first-line ART and switching to second-line ART were estimated. Crude and adjusted hazard ratios were calculated using the Cox regression model to identify risk factors associated with the two outcomes.

**Results:**

Of 23,248 adults and adolescents, 7,888 (34%) were tested for HIV viral load. The incidence rate of virological failure among those tested was 3.2 per 100 person-years follow-up and the rate of switching to second-line ART among all patients was 1.4 per 100 person-years follow-up. Factors associated with virological failure included: being adolescent; being lost to follow-up at least once; having WHO stage 3 and 4 at ART initiation; and having taken first-line ART elsewhere before coming to IHC. Of the 1032 patients who met virological failure criteria, 762 (74%) switched to second-line ART.

**Conclusions:**

We found high rates of virological failure among one third of patients in the cohort who were tested for viral load. Of those failing virologically on first-line ART, about one quarter were not switched to second-line ART. Routine viral load monitoring, especially for those identified as having a higher risk of treatment failure, should be considered in this setting to detect all patients failing on first-line ART. Strategies also need to be put in place to prevent treatment failure and to treat more of those patients who are actually failing.

## Introduction

Antiretroviral therapy (ART) has been available free-of-charge for more than 10 years in the public and private health sectors in Myanmar. By the end of 2015, 106,490 patients were on ART. This number accounted for 55% of the estimated People Living with Human Immunodeficiency Virus (PLHIV) in Myanmar. The National AIDS Program (NAP) has been rapidly scaling up ART in the country and aims to achieve universal access in a few years to reduce HIV-related morbidity and mortality [1]. With this rapid scaling up, it is also important to sustain treatment success with undetectable viral loads in patients on first-line ART. Otherwise, failing on first-line regimens can lead to a complicated, less tolerable and more expensive second-line ART regimen with fewer drug options if drug related toxicities develop. Therefore, it is important clinically and programmatically to learn more about the rate of first-line treatment failure, the rate of switching to a second-line ART regimen and to identify which patients are at risk in order to develop strategies to prevent developing of further failure cases.

Studies conducted in Asia and elsewhere have shown different rates (ranging from 1.1–4.5 per 100 person-years) and proportions (ranging from 11–28%) of patients failing on treatment [2–6], partly because treatment failure was diagnosed differently (clinically, immunologically or virologically) across these studies. The rate of patients switching to second-line ART has ranged from 2.2 to 3.3 per 100 person-years [7–11]. Studies have shown that different demographic, clinical and treatment factors were associated with treatment failure on first-line ART and switching to second-line ART [4,12–14]. However, there is a lack of published data in Myanmar on first-line ART failure and the rate of switching to a second-line regimen, both of which are important indicators for the Myanmar HIV/AIDS Program to assess.

The Integrated HIV Care (IHC) program, supported by The Union in Myanmar, has been providing treatment and care to PLHIV from all regions of the country since 2005. By 2015, nearly 30,000 patients were on ART and all patients’ data had been routinely collected in an electronic database. In this study, we retrospectively analysed the rates of treatment failure and switching to second-line ART in adolescent and adult patients receiving first-line ART in the IHC program. We also determined risk factors associated with these two outcomes.

## Methods

### Study design and study population

This study was a retrospective cohort analysis of all adolescent and adult PLHIV who were initiated on first-line ART under IHC care between 1^st^ February 2005 and 1^st^ July 2015. Adolescent (aged 10 to 19 years inclusive) and adult (older than 19 years) age groups are defined according to the WHO definition of age groups and populations in the 2013 HIV Consolidated guidelines [15]. We included ART naive patients as well as non-naïve patients who were previously on first-line ART at a private and government clinic/hospital. The following patients were excluded: i) women who were initiated on ART under the prevention of mother to child transmission (PMTCT) program; ii) patients who were already on second-line ART at the time of enrollment; and iii) patients whose duration of follow up was less than 6 months after ART had been initiated at the IHC.

### Study setting

#### Integrated HIV Care (IHC) program

Of a total of 15 regions in Myanmar, the IHC program has been operating in the Mandalay, Sagaing, Magway, Shan and Yangon regions through 33 sites (ART centres and decentralized sites) in collaboration with the National AIDS Control Program (NAP) since 2005. Patients attending IHC sites come from all 15 regions of Myanmar. The evolution of the IHC program has been described elsewhere [16]. Decentralized ART sites under the IHC program started in 2010 for patients who were identified as being stable on ART at ART centres and willing to receive treatment at decentralized sites. Health care providers at ART centres include specialist physicians, HIV clinicians and nurses. Decentralized sites do not initiate PLHIV on ART and do not have physicians able to care for ill and complicated patients. The latter are referred back to the ART centres for specialist care and advice.

#### Patient follow-up and care at IHC sites

The HIV management and ART provision at IHC sites follow the NAP and WHO treatment guidelines [15, 17]. Chronic HIV care including antiretroviral drugs and laboratory investigations are provided free of charge in all IHC sites. After ART initiation, patients are monitored for their treatment response clinically and immunologically. After having been on ART for six months or longer with increasing CD4 cell counts and good adherence to treatment, if a patient develops a new or recurrent WHO clinical stage 3 or 4 condition, clinical failure is suspected. Immunological failure is suspected if the CD4 cell count falls to the baseline or below or stays persistently below 100cells/μl after six months on ART. These clinical or immunological suspected failures are not routinely documented in the IHC database. The program follows a targeted viral load testing strategy as routine viral load monitoring is not available, but only some of the patients who have clinical and immunological failure on first-line ART are referred for HIV viral load testing by clinicians. Viral load testing using an automated c1000 RealTime PCR system (Bio-Rad Sciences, Hercules, California, USA and HIV Generic Charge Virale, Biocentric, Bandol, France) has been available in the Public Health Laboratory in Mandalay. This laboratory is under the management of the Ministry of Health, Myanmar, which has been supported by the Union since late 2012. Prior to this and since 2009, the samples were sent to an outside laboratory for viral load testing using Rotor-Gene Real-Time Analysis (PG. Biotech HIV detection kit). Before 2009, there was no viral load testing in the country and patients’ treatment was monitored clinically or immunologically.

Although reference is made to National and/or WHO HIV treatment guidelines, the diagnostic criteria of failure on first-line ART and the decision to switch to second-line ART differ from clinician to clinician. The routine practice is that if the viral load is detectable but is lower than the cut-off point for virological failure, the viral load test is repeated after 3 months of intensive adherence counselling. If the patient’s viral load becomes undetectable, or detectable but lower than the virological failure cut-off point, the first-line ART regimen is continued. If the patient’s viral load is higher than the cut-off point, a protease inhibitor based second-line ART regimen which is available in the program is prescribed. The virological failure cut-off point of the HIV viral load was 5000 copies/mL up to March 2015, and thereafter the threshold was reduced to 1000 copies/mL in line with recommendations in the WHO guidelines. Second-line ART (protease inhibitors) was only available from the end of 2008. Before this time, patients who were diagnosed as having ART failure on either clinical or immunological grounds were continued on first-line ART.

### Study variables

#### Outcome variables

The two outcome variables were virological failure while on first-line ART and switching to second-line ART. We defined virological failure as a viral load result greater than 5000 copies/mL before March 2015 and greater than 1000 copies/mL after March 2015. Switching to second-line ART was defined as a patient being switched to a protease inhibitor based ART regimen from a nucleoside reverse-transcriptase inhibitor and non-nucleoside reverse-transcriptase inhibitor based ART regimens. For those who switched to second-line ART, we also recorded the reason for switching as documented in the database.

#### Exposure variables

Exposure variables included: gender; age at ART initiation (groups included adolescents (10 to 19 years) [18] and adults (>19 years)); marital, employment and literacy status; HIV transmission risk factors; availability of a care taker (someone who comes along with the patient for clinic visit and helps patient for taking drugs at home, usually the patient’ family or close friend) when ART was initiated; baseline CD4 cell count and WHO clinical stage; first-line ART regimens at enrolment; history of ART before coming to IHC; baseline hepatitis B and C status; type of current IHC site (ART centre or decentralized site), number of times the patient had been documented as “lost to follow-up” from care; number of times the first-line ART regimen had been modified. First-line ART regimens that were modified included: tenofovir disoproxil fumarate (TDF) + lamivudine (3TC) + efavirenz (EFV); stavudine (d4T) + 3TC+ nevirapine (NVP); D4T+3TC+EFV; zidovudine (AZT) + 3TC+EFV; d4T+3TC+NVP; AZT+3TC+NVP; and TDF+3TC+NVP. We considered CD4 cell count measurements taken within three months either before or after ART initiation as the baseline CD4 cell count.

### Data source and data extraction

Data was extracted without the patient’s name and address from the electronic database of the IHC program which is routinely updated by data staff.

### Data analysis

Time to virological failure on first-line ART and switch to second-line ART, and the cumulative hazard of first-line failure and switching to second-line ART were estimatedusing Nelson-Aalen methods. Crude and adjusted hazard ratios with 95% confidence intervals were calculated for predictor variables to assess any association with virological failure and switching to second-line ART using the Cox proportional-hazards model. Predictor variables with a *P*-value of <0.2 in the univariate analysis were included in a multivariate regression model after excluding patients with missing data. A *P*-value of less than 0.05 was considered statistically significant. Probability of virological failure and switching to second-line ART were plotted.

The time to virological failure was defined as the time between the date of starting first-line ART and the date of the first diagnosis of virological failure. The time to switching to second-line ART was defined as the time between the date of starting first-line ART and the date of switching to second-line ART. For patients who died or were lost-to-follow-up or transferred out, the date of last appointment before the event was considered as the censor date. If a patient was lost to follow-up from the program for more than three months and then came back to the program, we considered that he/she was ‘lost to follow-up at-least once’. If a patient was lost to follow-up from care more than once, the date of the most recent lost to follow-up date was used as the censor date. For patients retained in care and without any event, 31^st^ December 2015 was considered the censor date. Statistical analyses were performed using Stata 12 [19].

### Ethics considerations

The study was based on a retrospective analysis of routinely collected data. Ethics approval from The Union Ethics Advisory Group (Paris, France) and permission from the National HIV/AIDS program, Department of Public Health, Ministry of Health and Sports, Myanmar were received for this study. Unique codes were used and all patient identifier information was removed before the analysis.

## Results

### Characteristics of the cohort

There were 23,248 adolescent and adult patients started on ART in the IHC program who met inclusion criteria of this study. They were followed up for a total of 70,689 person-years. Females accounted for 42% of the cohort and the median (Interquartile range) age at ART initiation was 36 (31–42) years. Adolescents constituted 3% of the cohort. The majority (55%) of the cohort was married. A few patients disclosed that they belonged to one of the key populations: men who had sex with men (1.4%); commercial sex workers (0.2%); and injection drug users (4%). Most of the patients (84%) were recorded as having a care taker. Overall, 15% of the patients had taken ART at private clinics, government hospitals and/or National AIDS program clinics before coming to the IHC sites. The most common starting ART regimens were: TDF + 3TC + EFV (36.5%); d4T+ 3TC + NVP (21.8%); and d4T+3TC+EFV (19.0%). The baseline prevalence of hepatitis B and C was 8% and 6.4% respectively. At the end of the study period, 30% of patients were receiving care at the decentralized ART sites and 13% of the cohort was recorded as being lost to follow-up at least one time. Half of the patients experienced ART regimen modification at least once during the study period (Table 1).

**Table 1:** Socio-demographic and clinical characteristics of patients who received first-line ART regimen at the Integrated HIV Care program, Myanmar, between 2005 and 2015.

Baseline socio-demographic characteristics		Number	%
All patients		23,248	100
Gender	Male	13,446	57.8
	Female	9,802	42.2
Age group (years)	10–19	633	2.7
	≥20	22,615	97.3
Marital status	Single	4,840	20.8
	Married	12,697	54.6
	Widowed	4,069	17.5
	Divorced/Separate	1,349	5.8
	Missing data	294	1.3
Employment status	Employed	16,619	71.5
	Not employed	6,192	26.6
	Missing data	438	1.9
Literacy	Literate	20,800	89.5
	Illiterate	2,214	9.5
	Missing data	235	1.0
Transmission risk	Heterosexual	19,329	83.1
	Men having sex with men	334	1.4
	Sex work	46	0.2
	Injecting drug use	941	4.0
	Blood transfusion	747	3.2
	Mother to child	488	2.1
	Missing	1,364	5.9
Care taker	Available	19,567	84.2
	Not available	3,681	15.8
Region of ART site	Mandalay	12,226	52.6
	Sagaing	2,255	9.7
	Magway	2,047	8.8
	Shan	2,924	12.6
	Yangon	3,796	16.3
Baseline clinical characteristics			
WHO staging	1 and 2	8,715	37.5
	3 and 4	14,533	62.5
CD4 count (cells/μl)	<100	7,647	32.9
	100–350	11,807	50.8
	>350	2,028	8.7
	Missing data	1,766	7.6
ART delivery site before enrolment	Private	2,372	10.2
	Public	783	3.4
	Unknown site	295	1.3
	Naïve	19,798	85.2
ART regimen	TDF+3TC+EFV	8,494	36.5
	AZT+3TC+EFV	2365	10.2
	d4T+3TC+EFV	4419	19.0
	d4T+3TC+NVP	5069	21.8
	AZT+3TC+NVP	2762	11.9
	TDF+3TC+NVP	13	0.1
	Missing	8	0.0
Hepatitis B	Positive	1,922	8.0
	Negative	19,597	84.0
	Missing data	1,729	7.0
Hepatitis C	Positive	1,480	6.4
	Negative	20,034	86.2
	Missing data	1,734	7.5
Follow-up clinical characteristics			
Current ART site	ART center	16,123	69.4
	Decentralized site	7,125	30.6
Loss to follow up (number of times)	0	20,305	87.3
	1	2572	11.1
	>1	371	1.6
Duration on ART (months)	6–12	3,574	15.4
	12–24	5,008	21.5
	>24	14,666	63.1
ART regimen modification (number of times)	0	11,511	49.5
	1–3	11,141	47.9
	>3	596	2.6

ART = antiretroviral therapy; WHO = World Health Organization; d4T = stavudine, AZT = zidovudine, 3TC = lamivudine, EFV = efavirenz, NVP = nevirapine, ABC = abacavir, TDF = tenofovir

### Virological failure

#### Incidence rate of virological failure

**Fig 1:**
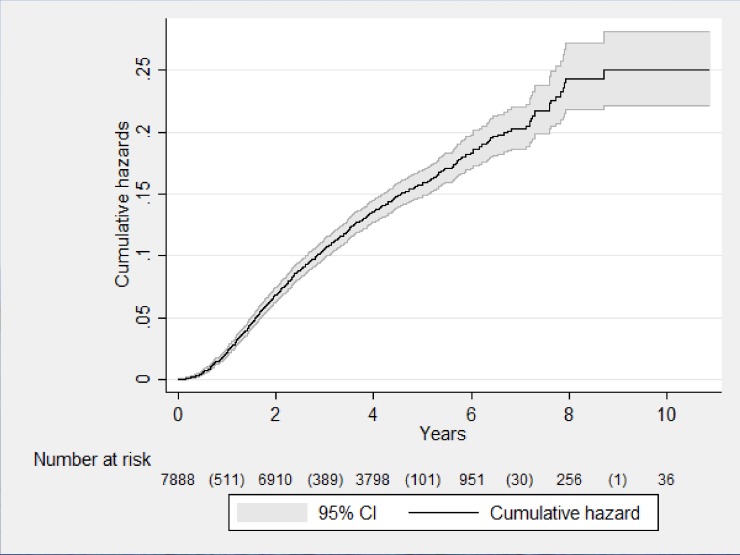
Nelson Aalen curve showing the cumulative hazard of virological failure in patients on first-line ART initiated at Integrated HIV Care program, Myanmar, between 2005 and 2015.

There were 7,888 (34% of the whole cohort) patients who were assessed for viral load, and of these 1032 (13%) met the virological failure criteria. The incidence rate of virological failure after first-line ART amongst those tested was 3.2 per 100 person-years of follow-up (PYFU). The cumulative hazard of virological failure at 5 years and 10 years after starting ART amongst those tested was 17% and 22% respectively (Fig 1).

Stratified by duration on first-line ART, the incidence rate of failure in patients who were on ART for less than one year, between one and two years and for more than two years was 65.9, 26.3 and 2.0 per 100 PYFU respectively.

#### Factors associated with virological failure

After adjusting the covariates, adolescent age at enrollment, patients with baseline WHO clinical stage 3 and 4 disease, patients with severe and moderate immune suppression with CD4 count less than 350 cells/μl, those who had taken ART at a private clinic before enrollment, and those who had a history of loss to follow-up from the care at least one time were all found to be at significantly higher risk of virological failure compared with reference groups. Patients who were divorced or separated from their spouses, who were receiving ART at decentralized sites and whose first-line ART regimen was modified more than one time had a significantly lower risk of failure. Gender, literacy, employment status, not having a care taker, the initial ART regimen and co-infection with Hepatitis B or C had no significant association with failure. (Table 2)

**Table 2:** Rates and predictors of virological failure in patients initiated on first-line ART in the Integrated HIV and Care Program in Myanmar between 2005 and 2015.

Socio-demographic factors		Number of virological failures	Rate per 100 PYFU^a^	Hazard Ratio (95% CI^b^)	p-value	Adjusted Hazard Ratio (95% CI^b^)	p-value
Total		1032	3.2				
Gender	Male	668	3.5	1.3(1.2–1.4)	<0.001	1.1(0.9–1.2)	0.56
	Female	364	2.7	ref^d^		ref	
Age group (years)	Adolescent (10–19)	64	10.4	3.3(2.5–4.2)	<0.001	4.2(2.0–8.8)	<0.001
	Adult (≥20)	968	3.1	ref		ref	
Marital status	Single	286	4.1	ref		ref	
	Married	542	3.1	0.8(0.7–0.9)	<0.001	0.9(0.8–1.1)	0.41
	Widowed	152	2.6	0.6(0.5–0.8)	<0.001	0.8(0.7–1.1)	0.23
	Divorced/Separate	32	2.2	0.5(0.4–0.8)	<0.01	0.6(0.4–0.9)	<0.05
	Missing	20	5.7	1.4(0.9–2.2)	0.17		
Employment status	Employed	738	3.2	ref			
	Not employed	284	3.4	1.1(0.9–1.2)	0.35		
Literacy	Literate	922	3.2	ref			
	Illiterate	100	3.6	1.1(0.9–1.4)	0.21		
Transmission risk	Heterosexual	846	3.1	ref		ref	
	Men sex with men	25	5.5	1.8(1.2–2.6)	<0.01	1.5(0.9–2.4)	0.06
	Sex work	2	4.1	1.3(0.3–5.4)	0.68	3.1(0.8–12.3)	0.11
	Injecting drug use	30	2.9	1.0(0.7–1.4)	0.80	0.9(0.6–1.4)	0.67
	Blood transfusion	36	3.4	1.1(0.8–1.5)	0.56	0.9(0.7–1.4)	0.85
	Mother to child	49	10.1	3.2(2.4–4.3)	<0.001	0.5(0.2–1.0)	0.06
Care taker	Available	807	3.4	ref		ref	
	Not available	225	2.8	0.8(0.7–0.9)	<0.05	1.0(0.8–1.2)	0.70
Baseline clinical factors							
WHO clinical stage	1 and 2	271	2.8	ref		ref	
	3 and 4	761	3.4	1.3(1.1–1.5)	<0.01	1.3(1.1–1.6)	<0.01
CD4 count (cells/μl)	<100	460	3.8	2.0(1.5–2.6)	<0.001	2.6(1.8–3.7)	<0.001
	100–350	408	2.7	1.5(1.1–1.9)	<0.05	2.0(1.4–2.8)	<0.001
	>350	36	1.9	ref		ref	
ART before enrolled	Private	255	5.5	2.0(1.7–2.3)	<0.001	2.2(1.8–2.5)	<0.001
	Public	55	2.9	1.03(0.8–1.4)	0.80	1.1(0.7–1.5)	0.77
	unknown site	17	4.6	1.6(0.9–2.5)	0.07	1.7(0.9–3.1)	0.07
	Naïve	705	2.8	ref		ref	
ART regimen	TDF+3TC+EFV	143	15.8	ref		ref	
	Other regimen^c^	885	2.8	0.2(0.1–0.2)	<0.001	0.9(0.7–1.1)	0.29
	Missing	4	20.2	1.2(0.5–3.3)	0.69		
Hepatitis B	Negative	892	3.1	ref		ref	
	Positive	90	4.3	1.4(1.2–1.7)	<0.01	0.9(0.7–1.3)	0.56
Hepatitis C	Negative	932	3.2	ref			
	Positive	50	2.8	0.8(.7–1.2)	0.33		
Follow-up clinical factors							
Current ART site	ART center	738	4.4	ref		ref	
	Decentralized site	294	1.9	0.4(0.4–0.5)	<0.001	0.5(0.4–0.6)	<0.001
Loss to follow up (number of times)	0	807	3.0	ref		ref	
	1	174	3.7	1.3(1.1–1.5)	<0.01	1.4(1.1–1.6)	<0.01
	>1	51	7.3	2.6(1.9–3.4)	<0.01	2.0(1.5–2.8)	<0.001
ART regimen modification (number of times)	0	493	15.8	ref		ref	
	1–3	504	1.9	0.1(0.1–0.1)	<0.001	0.1(0.1–0.2)	<0.001
	>3	35	1.7	0.1(0.1–0.1)	<0.001	0.1(0.1–0.2)	<0.001

Predictor variables with significant associations (p<0.2) in univariate analysis were adjusted in multivariate analysis. Missing data were excluded in the multivariate model. ART = antiretroviral therapy; WHO = World Health Organization; d4T = stavudine, AZT = zidovudine, 3TC = lamivudine, EFV = efavirenz, NVP = nevirapine, ABC = abacavir, TDF = tenofovir disoproxil fumarate

^a^ person-year follow up

^b^ confidence interval

^c^ other regimen included d4T+3TC+EFV, d4T+3TC+NVP, AZT+3TC+NVP, AZT+3TC+EFV, TDF+3TC+NVP, ABC+3TC+NVP/EFV

^d^ ref = reference group

### Switching to second-line ART

#### Incidence rate of switching to second-line ART

**Fig 2:**
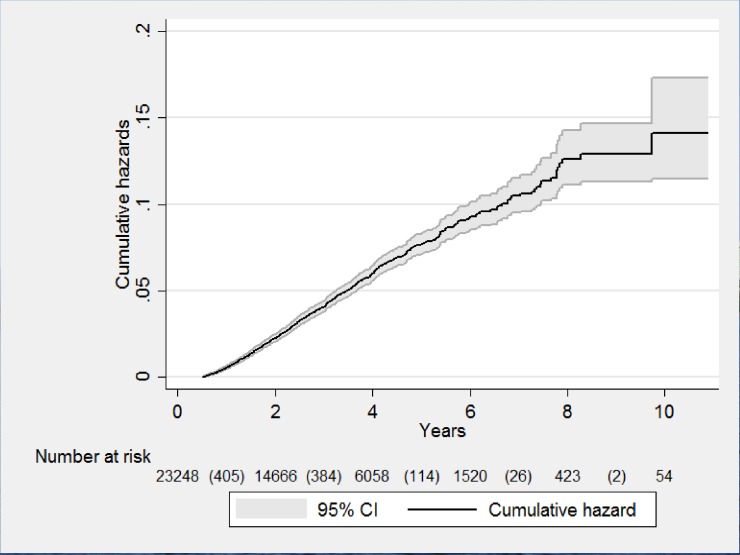
Nelson Aalen curve showing the cumulative hazard of switching to second-line ART after starting first-line ART at the Integrated HIV Care program, Myanmar, between 2005 and 2015

Of 23248 patients, 931 (4%) patients switched to second-line ART with a rate of 1.35 per 100 PYFU. The cumulative hazard of switching to second-line ART at 5 years of taking first-line ART was 7% and at 10 years was 12% (Fig 2). Of 931 patients who switched to second-line ART, 88% were switched due to treatment failure, 1.3% due to toxicity and 10% had no recorded information.

Stratified by duration on first-line ART, the incidence of switching to second-line ART in patients who were on ART for less than one year, between one and two years and more than two years was 4.49, 3.91 and 0.89 per 100 PYFU respectively.

#### Factors associated with switching to second-line ART

After adjusting the covariates, adolescent age at enrollment, patients with baseline WHO clinical stage 3 and 4 disease, those with severe and moderate immune suppression with CD4 counts less than 350 cells/μl, those who had taken ART at private and public clinics before enrollment, those whose first ART regimens were not TDF+3TC+EFV and those who had a history of loss to follow-up from the care at least one time were significantly associated with a higher risk of switching to second-line ART.

Patients who were receiving ART at a decentralized site and whose first-line ART regimen were modified more than one time were at significantly lower risk of switching to second-line ART. Patients’ gender, marital, employment and literacy status, having no care taker at initiation of ART and co-infection with hepatitis B or C had no significant association with changing to second-line treatment. (Table 3.)

**Table 3:** Rates and predictors of switching to second-line ART in patients on first-line ART, Integrated HIV and Care Program, Myanmar, between 2005 and 2015.

Socio-demographic factors		Number of switches to second-line ART (%)	Rate per 100 PYFU^a^	Hazard Ratio (95% CI^b^)	p-value	Adjusted Hazard Ratio (95% CI^b^)	p-value
Total		931	1.35				
Gender	Male	584	1.47	1.2(1.1–1.4)	<0.01	1.0(0.8–1.1)	0.68
	Female	347	1.19	ref^d^		ref	
Age group (years)	Adolescent (10–19)	46	2.78	2.2(1.6–2.9)	<0.001	2.7(1.1–6.4)	<0.05
	Adult (≥20)	885	1.32	ref		ref	
Marital status	Single	241	1.66	ref		ref	
	Married	503	1.34	0.8(0.7–0.9)	<0.01	0.9(0.8–1.0)	0.44
	Widowed	142	1.15	0.7(0.6–0.8)	<0.001	0.8(0.6–1.0)	0.09
	Divorced/Separate	32	0.93	0.6(0.4–0.8)	<0.01	0.7(0.5–1.0)	0.08
Employment status	Employed	651	1.33	ref			
	Not employed	272	1.46	1.1(0.9–1.3)	0.21		
Literacy	Literate	834	1.36	ref			
	Illiterate	90	1.33	1.0(0.8–1.2)	0.72		
Transmission risk	Heterosexual	769	1.32	ref		ref	
	Men sex with men	20	2.05	1.6(1.0–2.4)	0.05	1.2(0.7–2.0)	0.44
	Sex work	1	0.78	0.6(0.1–4.3)	0.61	0.6(0.1–4.4)	0.63
	Injecting drug use	28	1.26	1(0.7–1.5)	0.87	1.0(0.7–1.5)	0.95
	Blood transfusion	36	1.67	1.3(0.9–1.8)	0.15	1.1(0.8–1.6)	0.47
	Mother to child	38	2.93	2.3(1.7–3.2)	<0.001	0.8(0.3–1.9)	0.58
Care taker	Available	717	1.33	ref			
	Not available	214	1.45	1(0.9–1.2)	.75		
Baseline clinical factors							
WHO staging	I/II	235	1.00	ref		ref	
	III/IV	696	1.54	1.5(1.3–1.8)	<0.001	1.5(1.3–1.8)	<0.001
CD4 count (cells/μl)	<100	415	1.81	2.9(2.1–4.0)	<0.001	3.0(2.0–4.4)	<0.001
	100–350	377	1.11	1.8(1.3–2.5)	<0.001	5.5(3.7–8.1)	<0.001
	>350	32	0.64	ref)			
ART before enrolled	Private	256	3.33	3(2.6–3.5)	<0.001	4.2(3.6–5.0)	<0.001
	Public	57	1.65	1.4(1.1–1.9)	0.01	1.7(1.2–2.5)	<0.01
	Unknown site	15	1.84	1.7(1.0–2.9)	<0.05	1.9(1.0–3.5)	<0.05
	Naïve	603	1.06	ref		ref	
ART regimen	TDF+3TC+EFV	133	0.93	ref		ref	
	Other regimen^c^	795	1.45	1.2(1.0–1.4)	0.08	3.4(2.7–4.3)	<0.001
Hepatitis B	Negative	787	1.33	ref		ref	
	Positive	94	1.68	1.3(1.0–1.6)	<0.05	1.3(1.0–1.6)	<0.05
Hepatitis C	Negative	841	1.38	ref			
	Positive	41	1.08	0.8(0.6–1.1)	0.21		
Follow-up clinical factors							
Current ART site	ART center	666	1.62	ref			
	Decentralized site	265	0.96	0.5(0.5–0.6)	<0.001	0.5(0.4–0.6)	<0.001
Lost to follow-up (number of times)	0	739	1.28	ref		ref	
	1	156	1.65	1.2(1.0–1.5)	<0.05	1.3(1.0–1.6)	<0.01
	>1	36	2.27	1.7(1.2–2.4)	<0.01	1.5(1.0–2.1)	0.04
ART regimen modification (number of times)	0	499	2.27	ref		ref	
	1–3	404	0.93	0.3(0.3–0.3)	<0.001	0.2(0.2–0.2)	<0.001
	>3	28	0.88	0.3(0.2–0.4)	<0.001	0.1(0.1–0.2)	<0.001

Predictor variables with significant associations (p<0.2) in univariate analysis were adjusted in multivariate analysis. Missing data were excluded in the multivariate model. ART = antiretroviral therapy; WHO = World Health Organization; d4T = stavudine, AZT = zidovudine, 3TC = lamivudine, EFV = efavirenz, NVP = nevirapine, ABC = abacavir, TDF = tenofovir disoproxil fumarate

^a^ person-year follow up

^b^ confidence interval

^c^ other regimen included d4T+3TC+EFV, d4T+3TC+NVP, AZT+3TC+NVP, AZT+3TC+EFV, TDF+3TC+NVP, ABC+3TC+NVP/EFV

^d^ ref = reference group

### Gap between virological failure and switching to second-line ART

Of 1032 patients who had a detectable viral load and met the criteria for treatment failure, 762 (74%) switched to second-line ART. Of 270 patients who did not switch to second-line ART during study period, 103 (37%) died or were recorded as lost to follow up and 159 (60%) were on first-line ART and still in care at the end of the study period. The gaps between virological failure and switching to second-line ART were highest in adolescents, those whose risk of HIV transmission was from the mother, those who were initiated with TDF+3TC+EFV regimens and those who had never modified their first-line ART regimen (Tables 1 and 2).

## Discussion

This study of adolescent and adult patients on ART in Myanmar provides important findings. First, the overall rate of treatment failure was 3.2 per 100 PYFU and the rate of switching to second-line ART was 1.35 per 100 PYFU. The 10 year cumulative incidence of failing on first-line ART was 22% and of switching to a second-line protease inhibitor based ART regimen was 12%. These findings highlight the gap between people failing on treatment and people switching treatment due to treatment failure. The rate of switching to second-line ART might be higher if all patients on first-line treatment can get access to viral load testing. These findings are also of value in estimating the needs of second-line ART in the country and in planning the necessary care for patients who are on second-line ART.

Second, this study shows that the virological failure rate in Myanmar is higher than the virological failure rate reported in other studies: from China (12% failed after 2 years on ART)[20], from South Africa (failure rate of 4.5 per 100 PYFU)[6], from Cambodia (11.6% failed with a viral load cut-off of 400 copies/mL and 4.3% with a viral load cut-off of 30,000 copies/mL)[2] and from a pooled estimate for the Asia region from a recent systematic review [21]. However, comparisons of rates of failure across different studies and countries are difficult because of different viral load cut-off points, duration of time on ART and follow-up periods.

Third, the rate of switching to second-line ART in this study (1.3 per 100 PYFU) is comparable to results of some other studies, especially from programs which have no routine viral load monitoring. However, the incidence of switching to second-line ART in our study was lower than that reported from other programs which do have routine viral load monitoring.[5,7,8] These findings strongly suggest that there is a possibility of under-diagnosis of treatment failure in our program and there may be substantial numbers of patients with virological failure who need to switch to second-line ART. Therefore, routine viral load monitoring should be considered in all program settings if resources permit [22].

We found that the incidence of virological failure and switching to second-line ART was highest in the first year after ART had been initiated at the IHC. This finding contrasts with other reported findings that showed that viral suppression was usually higher in the first two years after treatment compared to later years when ART failure started to increase [8,23]. One of the likely reason might be due to the fact that access to viral load testing was significantly higher among recent cohorts of patients (6–12 months) compared to others. Hence the recent cohort of patients were more likely to get diagnosed as virological failure earlier and contributing less number of person-years of follow-up. Whereas older cohorts were less likely to get viral load tested and contributing to more person-years of follow-up. Another reasons for this discrepancy might be selection bias as half of the patients in the group categorized as “one year treatment duration (6–12 months)” in our study had taken ART previously at private clinics before coming to IHC. More in-depth analysis and research is needed to understand the association between duration on ART and the treatment success in this population. Nonetheless, this finding is still useful to inform the program about the need for careful monitoring of patient adherence and response to treatment in the initial two years of first-line ART.

Adolescents were at higher risk of treatment failure and of switching to second-line ART compared with adults. Despite the higher switching rate, the adolescent group still had a large gap in being able to access second-line ART when they failed on first-line ART. This merits further research and also suggests that health care providers need to consider customized services for this age group with respect to drug adherence and clinical care due to the challenges that they are known to face [24,25].

Starting ART at an advanced stage of illness or immunosuppression was associated with treatment failure, a finding that is in line with many other studies [2,6,13]. Patients who had taken ART at private clinics or other facilities before being enrolled to IHC also had a higher risk of ART failure, maybe because they received a non-standardized regimen or suboptimal therapy in the other facilities. Thus, it is worth considering the need for viral load or resistance testing before starting on a standardized regimen at the IHC clinic in this group [26].

There is published evidence that patients who miss a clinic visit, who are not 100% retained in care or who have poor drug adherence are at higher risk of virological failure [13,27,28]. We observed that being lost to follow up at least once was associated with treatment failure in this study which indicates that a patient lost from care is a proxy for suboptimal adherence and interruption of therapy. This group of patients needs to be further assessed by social workers or counsellors in terms of understanding why they are lost to care and appropriate support should be provided.

We also found that people who started with TDF+3TC+EFV were less likely to switch to a second-line regimen. This may be due to the fact that TDF+3TC+EFV can be taken as a once-daily regimen. In addition, this regimen can be less toxic and therefore better tolerated than other regimens. Hence, patients who started with TDF+3TC+EFV regimen may have better adherence and be less likely to fail.

The rate of virological failure and switching to second-line was not significantly different between patients who had a caretaker at the first visit and those who did not have a caretaker. As having a caretaker is one of the criteria necessary to start ART in this program, we need to reconsider the care taker requirement which may not be necessary for starting ART.

One of the most important findings was that not all virologically failing patients were switched to protease inhibitor based second-line ART, especially the group of adolescents. We cannot certain that all patients with virological failure need to switch to alternate treatment regimens. Because there is no drug resistance testing available in our setting to inform whether the detectable viral load is due to resistance to first-line ART or non-adherence to medication. However, the evidence from other studies shows that about 90% of the patients with detectable viral load are already resistant to first-line ART in these circumstances where there is no routine system for doing viral load monitoring [29].

Another important observation from this study is that one in three of those who did not switch to second-line ART died or were lost to follow-up. Previous studies have shown that a delay in treatment switching when required is associated with high mortality and lost to follow-up [5,9,10]. Hence, it is important for the program to follow-up those patients who have not switched but are still under care.

One of the strengths of this study is the relatively large sample size with individual patient follow-up time data. The study was also conducted within the routine program setting which reflects ground reality. Because of the existing mechanisms of data validity, the data are relatively good and reliable. We also reported this study according to STROBE guidelines [30].

However, there were several limitations to this study. First, we excluded: 1) women who entered the program for PMTCT interventions as the database does not fully capture their starting ART regimens; 2) 40 patient records with missing ART start date and/or the type of regimen; 3) 6778 patients with follow-up less than 6 months. Second, although clinical and immunological failure was used as a proxy for virological failure by clinicians for deciding to switch to second-line ART or to refer for a HIV viral load test, there was no data about how many patients had clinical and immunological failure in this program. Moreover, not all viral load measurements were captured because some patients died or were lost to follow-up after testing and before the results were dispatched to be recorded in the patient’s file. This may affect the rate of failure observed in this study. Third, as this study was an analysis of a large cohort followed up for 10 years, the viral load cut off points to declare virological failure differed over these years. Fourth, there was no viral load testing or available second-line ART from 2005 to 2008 and patients who experienced virological failure during these four years were not be able to be diagnosed in the program or captured in this database. Fifth, there was no information about whether patients with virological failure were developing resistance to their current ART or were having viral blips which are important factors to decide upon when therapy switches are being considered [31]. Sixth, there were missing data and there may also have been data recording and data entry errors, as our analysis was based on routinely collected program data. Finally, we could not answer the reasons for why some of those who failed did not switch to second-line ART.

## Conclusions

From this observational study of a large cohort of patients on ART in Myanmar, we found a high incidence of virological failure amongst patients who were tested for viral load. There was also a gap in switching to second-line ART in patients who had failed on first-line ART, especially amongst adolescents. The true incidence of virological failure and the exact number of patients who need to switch to second-line ART cannot be determined due to scarcity of viral load testing and lack of capacity to do drug resistance testing. Routine testing of viral load, as WHO has recommended, in this setting can help to diagnose patients who fail on their treatment especially those who are adolescents, who have been lost to follow-up at least once, with advanced stage at ART initiation and who have taken ART elsewhere before coming to IHC. Further research needs to be considered to better understand those who are at high risk of treatment failure. This will help the development of strategies to prevent treatment failure and to plan for resources to ensure that those actually failing can be properly treated.

## Supporting information

S1 Dataset First-line ART failure and switching to second-line ART, Myanmar. (DTA)
